# Triboelectrification‐Enabled Self‐Powered Data Storage

**DOI:** 10.1002/advs.201700658

**Published:** 2018-01-05

**Authors:** Shuang Yang Kuang, Guang Zhu, Zhong Lin Wang

**Affiliations:** ^1^ Beijing Institute of Nanoenergy and Nanosystems Chinese Academy of Sciences Beijing 100083 P. R. China; ^2^ Institute of Semiconductors Chinese Academy of Sciences Beijing 100083 P. R. China; ^3^ University of Chinese Academy of Sciences Beijing 100049 P. R. China; ^4^ CAS Center for Excellence in Nanoscience National Center for Nanoscience and Technology (NCNST) Beijing 100083 P. R. China; ^5^ School of Materials Science and Engineering Georgia Institute of Technology Atlanta GA 30332 USA

**Keywords:** computer science, data storage, nanoenergy, nanogenerators, triboelectrification

## Abstract

Data storage by any means usually requires an electric driving power for writing or reading. A novel approach for self‐powered, triboelectrification‐enabled data storage (TEDS) is presented. Data are incorporated into a set of metal‐based surface patterns. As a probe slides across the patterned surface, triboelectrification between the scanning probe and the patterns produces alternatively varying voltage signal in quasi‐square wave. The trough and crest of the quasi‐square wave signal are coded as binary bits of “0” and “1,” respectively, while the time span of the trough and the crest is associated with the number of bits. The storage of letters and sentences is demonstrated through either square‐shaped or disc‐shaped surface patterns. Based on experimental data and numerical calculation, the theoretically predicted maximum data storage density could reach as high as 38.2 Gbit in^−2^. Demonstration of real‐time data retrieval is realized with the assistance of software interface. For the TEDS reported in this work, the measured voltage signal is self‐generated as a result of triboelectrification without the reliance on an external power source. This feature brings about not only low power consumption but also a much more simplified structure. Therefore, this work paves a new path to a unique approach of high‐density data storage that may have widespread applications.

Data storage refers to a process in which information is recorded into and retrieved from a media. Modern means of high‐density data storage primarily fall into three categories, i.e., magnetic data storage, optical data storage, and electrical data storage.^1^, ^2^, ^3^, ^4^, ^5^, ^6^, ^7^, ^8^, ^9^, ^10^, ^11^ Taking the example of hard drive, CD/DVD, and USB flash disk, consumer products based on the above three mechanisms are playing indispensable roles in our daily life. Considerable research efforts are still being made to realize high‐density data storage devices at low cost by either improving the current technologies or exploring new approaches.^12^, ^13^, ^14^, ^15^, ^16^ Ross et al. reported high‐density magnetic storage media by making magnetic pillars, pyramids, and dots over large area with periods of 100–200 nm.^17^ Pohl et al. developed near‐field optical data storage, which indicates a resolution of at least λ/20 compared with the values of λ/2.3 for conventional optical microscopy.^18^ However, both magnetic and optical storage still suffer from the problem of storage density limitation, which is attributed to superparamagnetic effect^19^, ^20^ and optic diffraction,^21^, ^22^ respectively. Besides, the reliance on external power supply, high cost, and complicated fabrication of storage materials are concerns for the development of data storage technology.

In this work, we present a novel approach of triboelectrification‐enabled data storage (TEDS) without supplying an external power. Data writing is realized by incorporating American Standard Code for Information Interchange (ASCII) into a set of metal‐based patterns. As a probe slides across the patterned surface, triboelectrification between the scanning probe and the patterns produces alternatively changing electric potential in quasi‐square wave. The trough and crest of the quasi‐square wave signal are coded as binary bits “0” and “1,” respectively, while the time span of the trough and the crest is associated with the number of bits. Information of letters and sentences is stored through either square‐shaped or disc‐shaped surface patterns. Real‐time data retrieval is demonstrated with the assistance of software interface. Based on experimental data and numerical calculation, the theoretically predicted maximum data storage density could reach as high as 38.2 Gbit in^−2^. For the TEDS reported in this work, the measured voltage signal is self‐generated as a result of the triboelectrification without the reliance on an external power source, this kind of self‐powered system has been broadly reported in previous researches.^23^, ^24^, ^25^, ^26^, ^27^ This feature brings about not only low power consumption but also a much more simplified structure. Moreover, the surface triboelectrification enables a thin‐film setup that benefits miniaturization as well as storage density. Besides, materials selection is greatly expanded to a much broader scope as compared to conventional techniques. Therefore, this work paves a new path to a fundamentally unique approach to high‐density data storage that may have widespread applications.

The basic structure of the TEDS is diagramed in **Figure** **1**a, which mainly has two components. The underlying part constructed on a polymethylmethacrylate (PMMA) substrate has surface patterns into which meaningful data are incorporated. The patterned network is generated through mask‐assisted deposition of copper on a thin film made of polytetrafluoroethylene (PTFE). The other part is a core–shell structured probe that scans across a column of patterns. It has a metallic inner core made of copper and an electrification layer made of PTFE as a coating shell. A mechanical arm connects the probe with an electric motor that determines the scanning path of the probe. The detailed fabrication process and experimental setup are discussed in the Experimental Section. As the probe scans, an open‐circuit voltage (*V*_oc_) or the electric potential difference between the probe and the copper network is measured. Because of the triboelectrification on the contact surface between the two dissimilar materials, charge transfer takes place between the contacted surfaces.^28^, ^29^, ^30^ As extensively reported in previous works, negative triboelectric charges are generated on the electrification layer of the probe, while positive ones appear on the copper‐based patterns.^31^, ^32^, ^33^ In the open‐circuit condition, the measured voltage is only dependent on the position of the probe and the surface charge density on the electrification layer.^34^, ^35^ Figure 1b illustrates the process as the probe scans across a metal strip. At the state “I,” excessive negative triboelectric charges on the probe reduce the electric potential of the inner core, while the extra positive triboelectric charges on the metal strip increase the electric potential of the metal strip. As a result, the measured *V*_oc_ has the maximum value. When the probe slides onto the metal strip, the negative triboelectric charges are partially screened by their counterparts of the opposite sign, which yields the reduced *V*_oc_ (i.e., state “II” in Figure 1b). Once the probe completely overlaps with the metal strip, the triboelectric charges are completely screened; thus, the *V*_oc_ vanishes (i.e., state “III” in Figure 1b). *V*_oc_ then grows again as the probe slides off the metal region (i.e., state “IV” in Figure 1b). This process can be quantitatively described by an analytical model. If the cross section of the probe is square shaped (illustrated in Figure 1c), the measured *V*_oc_ can be analytically expressed by the following Equation (1). The detailed derivation is presented in Note S1 (Supporting Information)

(1) $$V_{OC}\left(t\right)\;\;=\;\;\;\{\begin{array}{c} c\sigma_{0}\left(\frac{v}{d}t-1\right)\;\;\;+\;\;V_{0},\;0<t\leq \frac{d}{v}\\ V_{0},\;\frac{d}{v}<t\leq \frac{D}{v}\\ c\sigma_{0}\left(\frac{D}{d}-\frac{v}{d}t\right)\;\;\;+\;\;V_{0},\;\frac{D}{v}<t\leq \frac{D+d}{v}\\ -c\sigma_{0}+V_{0\;},\;t>\frac{D+d}{v} \end{array}$$

where σ_0_ is the surface charge density on the probe, *V*_0_ is the reference voltage, *d* is the lateral length of the probe, *D* is the lateral length of a unit pattern, *v* is the sliding velocity of the probe, and *c* is a proportional constant, and *t* is the time, assuming *t* = 0 is the initial instant of the process.

**Figure 1:**
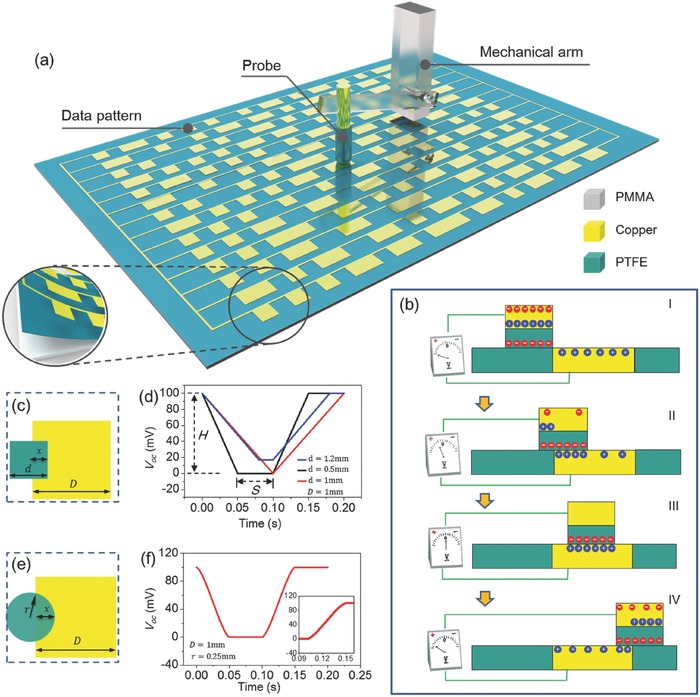
The setup of the TEDS and its working principle. a) Structure of the TEDS with the strip‐shaped surface patterns. b) Charge distribution as the probe scans across a unit pattern. c) Top‐down view of a square‐shaped probe and a unit pattern. d) Analytically calculated voltage signal. e) Top‐down view of a round‐shaped probe and a unit pattern. f) Analytically calculated voltage signal.

The function curves of Equation (1) are plotted in Figure 1d. If the lateral length of the probe *d* is smaller than that of the unit pattern*D*, for example, *d* = 0.5 mm <*D* = 1 mm, a sloped quasi‐square wave can be observed, as shown in Figure 1d. Two parameters are defined here for more detailed analysis. One is the amplitude of the wave (labeled as *H* in Figure 1d). From Equation (1) we can deduce *H* = −*cσ*_0_; the other is the time span of the wave trough (labeled as *S* in Figure 1d), which can be deduced as *S* = (*D* − *d*)/*v*. As the probe's size enlarges, the wave becomes sharpened until the probe and the unit pattern are equally sized (*d* = *D* = 1 mm, Figure 1d). Further increasing of the probe size yields reduced *H* (*d* = 1.2 mm >*D* = 1 mm, Figure 1d). *H* and *S* are key parameters in the following data recognition, which are discussed in Note S1 (Supporting Information). If the cross section of the probe is round shaped, as illustrated in Figure 1e, the analytically calculated *V*_oc_ is presented in Figure 1f. The detailed derivation is discussed in Note S1 (Supporting Information). The curve in Figure 1f reveals that the edge of the wave trough becomes rounded, as illustrated by the enlarged inset in Figure 1d.

Alternatively, the voltage signal *V*_oc_ can be calculated by numerical simulation via COMSOL. To elaborate how data are stored and retrieved, we selected a section of strip‐shaped patterns for illustration, as shown in **Figure** **2**a. All of the individual metal patterns and the intervals in between have integral numbers of a unit size (1 mm). The COMSOL model in Figure 2b is built accordingly with detailed settings discussed in the Experimental Section. As the probe slides across the patterns at a sliding velocity of 1 mm s^−1^, the electric potential distribution is presented in a series of sequential states. As the probe slides, the *V*_oc_ variation, i.e., the change of the electric potential difference between the probe and the metal network, is shown in Figure 2c, which also yields a sloped quasi‐square wave. The five points marked on the curve from “I” to “V” are associated to the five states in Figure 2b, respectively. Correspondingly, the experimentally measured *V*_oc_ signal when a probe slides across the patterns in Figure 2a is exhibited in Figure 2d, which is in great consistence to the calculated result in Figure 2c.

**Figure 2:**
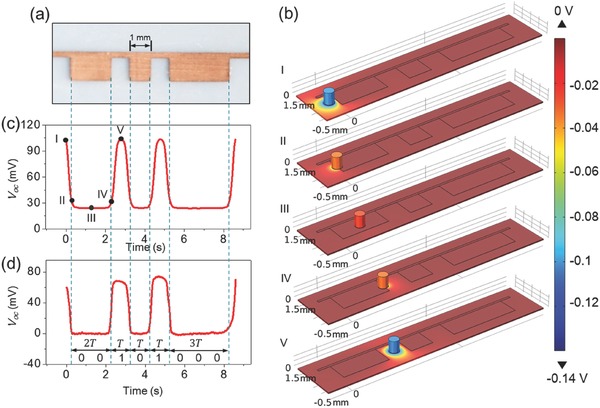
The simulation results of the TEDS via COMSOL. a) Photograph of a string of strip‐shaped patterns for data storage. b) Electric potential distribution as the probe scans across the pattern in five sequential states. c) Simulated *V*_oc_ as the probe scans across the patterns in Figure 2b. d) Experimentally measured *V*_oc_ as the probe scans across the patterns in Figure 2a.

As for data reading, the trough and the crest of the quasi‐square wave signal are interpreted as “0” and “1,” which correspond to the metal region and the interval area, respectively. The number of bits is determined by the time span of the crest and the trough. Because the probe moves at a constant velocity, the time span has integral numbers of a unit period “*T*.” Explicitly marked in Figure 2d, the leftmost metal region comes along with the first trough of the wave signal that has a time span 2*T*. As a result, this section of the pattern is then coded as “00.” For the similar reasoning, the rest of the patterns yields codes of “1,” “0,” “1,” and “000.” If put together, a string of codes “00101000” is then generated, which corresponds to a character “(” according to ASCII. Following the above process, data are then retrieved by interpreting the obtained voltage signal.

To further demonstrate the data storage process, a complete string of patterns was fabricated, which is diagramed in **Figure** **3**a. The feature size of the unit pattern is 2 mm, as shown in the inset in Figure 3a. Scanned by a probe with a diameter of 0.4 mm, the obtained voltage signal (measured by Keithley 6514, Tektronix) after baseline drift compensation is displayed in Figure 3b. The retrieved bit codes are also labeled in Figure 3b. Based on the ASCII, the translated information after decoding is “Data Storage” with each character corresponding to a highlighted section in Figure 3b. The feature size of a unit pattern can be further scaled down to 1 mm to promote the data storage density, as shown in Figure 3c. For the same reasoning, the phrase “BINN welcome you” can be retrieved from the generated voltage signal in Figure 3d. Besides strip‐shaped patterns, the data can be incorporated into disc‐shaped patterns that are normally adopted in optical and magnetic storage. The arc length of a unit pattern in **Figure** **4**a,b is 2 and 1 mm, respectively. As the probe scans across the patterns in a circular motion, the obtained voltage signal from the above two sets of patterns is shown in Figure 4c,d, respectively. The first set yields a phrase “Data Storage” (Figure 4c), while the second set results in a word “Triboelectrification” (Figure 4d). Therefore, the approach in this work proves to be compatible to currently used data storage configuration, in which a data disc spins while a probe shifts in the radial direction.

**Figure 3:**
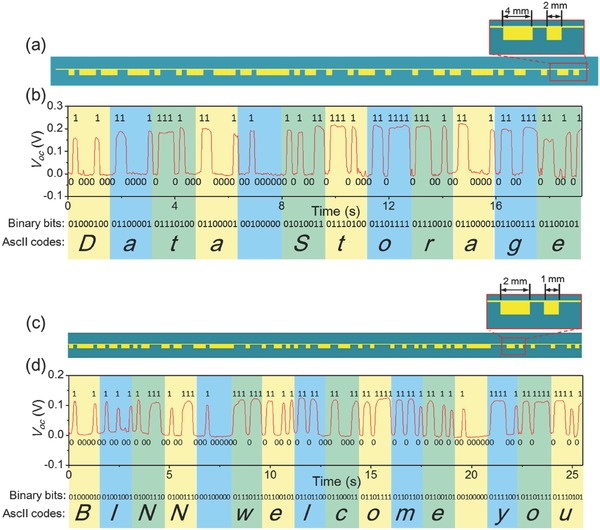
Two types of strip‐shaped TEDS with different storage density. a) Schematic diagram of the strip‐shaped TEDS with the unit pattern size of 2 mm. b) Measured *V*_oc_ signal and corresponding retrieved information from the TEDS in Figure 3a. c) Schematic diagram of the strip‐shaped TEDS with the unit pattern size of 1 mm. d) Measured *V*_oc_ signal and corresponding retrieved information from the TEDS in Figure 3c.

**Figure 4:**
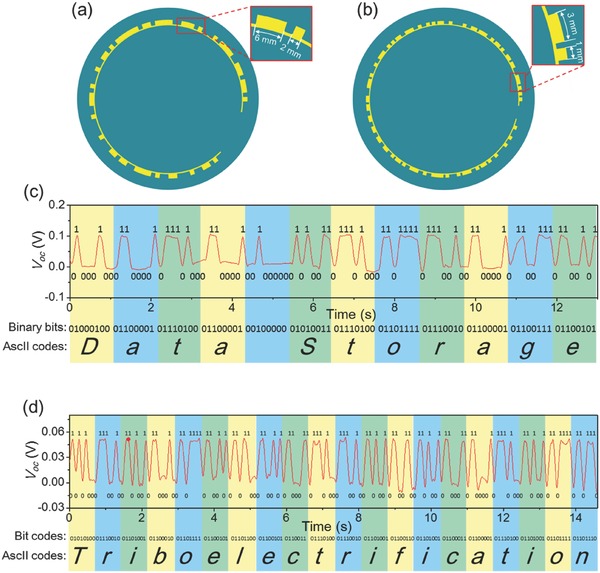
Two types of disc‐shaped TEDS with different storage density. a) Schematic diagram of the disc‐shaped TEDS with the unit pattern size of 2 mm. b) Schematic diagram of the disc‐shaped TEDS with the unit pattern size of 1 mm. c) Measured *V*_oc_ signal and corresponding retrieved information from the TEDS in Figure 4a. d) Measured *V*_oc_ signal and corresponding retrieved information from the TEDS in Figure 4b.

In this work, the maximum data storage density of the TEDS is discussed based on numerical simulation via COMSOL. For the obtained quasi‐square wave signal, the binary bits “0” and “1” are represented by the trough and the crest of the wave, respectively. So, the amplitude of the wave, i.e., the value of *H* in Figure 1d, directly affects the data reading. The factors that are associated with the value of *H* are first investigated via COMSOL simulation based on the model in **Figure** **5**a. To explore the effect of the electrification layer thickness, the unit pattern size *D* was set to be 1 mm, and the surface charge density on the probe σ was set to be 1 µC m^−2^. As the thickness *t* shrinks from 1.2 to 0.01 mm, the simulated values of *H* are plotted in Figure 5b. The fitted function expression of the curve in Figure 5b was found to be

(2) $$H\left(t\right)=\frac{1}{0.49+1.8\;\times 10^{3}t+0.94\;\times 10^{6}t^{2}}$$

**Figure 5:**
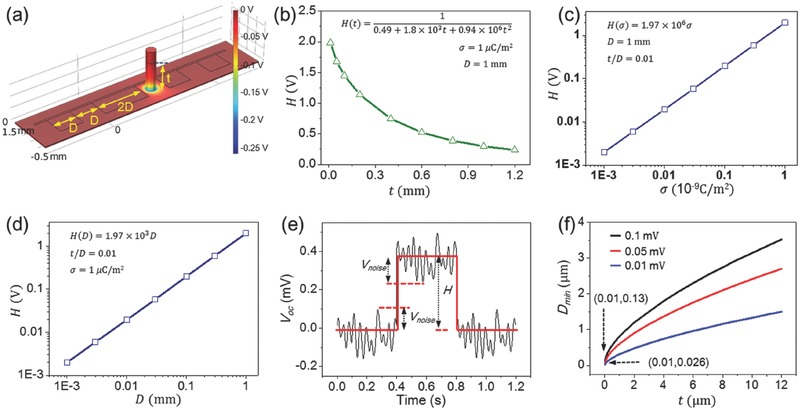
Storage density limit analysis by COMSOL simulation. a) Simulation model for calculating the storage density limit. b) The simulated result of the relation between *H* and *t* at the certain values of *D* and σ. c) The simulated result of the relation between *H* and σ at the certain values of *D* and *t*/*D*. d) The simulated result of the relation between *H* and *D* at the certain values of σ and *t*/*D*. e) Background noise of the measured voltage signal. f) The minimum unit pattern size determined by the background noise and thickness of the electrification layer.

The fitting process can be found in Note S2 (Supporting Information). If *D* is considered to be a variable, a series of simulations reveal that it is the ratio of *t* to *D* instead of just *t* that determines the expression of *H*, which follows the function below

(3) $$H\left(t/D\right)=\frac{A}{0.49+1.8t/D+0.94{\left(t/D\right)}^{2}}$$

where *A* is only determined by *D* and σ. At a set *t*/*D* and *D* (e.g., *t*/*D* = 0.01 and *D* = 1 mm in Figure 5c), *H* as well as *A* is found to have a linear relationship with σ, as presented by the fitted function in Figure 5c. Similarly, provided with a set t/*D* and σ (e.g., *t*/*D* = 0.01 and σ = 1 µC m^−2^ in Figure 5d), *H* and *A* are both linearly associated with *D*, which is illustrated in Figure 5d. Without the loss of generality, *H* can be expressed as

(4) $$H\left(\sigma ,D,t/D\right)=\frac{A\left(\sigma ,D\right)}{0.49+1.8t/D+0.94{\left(t/D\right)}^{2}}$$

Combining the results in Figure 5b–d, it can be obtained that

(5) $$H(\sigma ,D,t/D)=\frac{k\sigma D}{4.4+16.2t/D+8.5{\left(t/D\right)}^{2}}$$

where *k* = 9 × 10^9^ Nm^2^ C^−2^. At the certain value of *t* and *D*, *H* = *aH* (σ) = *cσ*, *a* and *c* are both constant, which is in complete accordance with the analytical theory results in Figure 1d,f.

The maximum data storage density can be derived from the minimum size of the unit pattern, i.e., *D_min_*. Considering the background noise of the signal acquisition system, i.e., *V*_noise_ in Figure 5e, the criteria for identifying the correct voltage signal are *H* > 2*V*_noise_. As a result, the limit of the data storage density occurs at

(6) $$H\left(\sigma ,D_{min},t/D_{min}\right)=2V_{\mathrm{noise}}$$

By submitting Equation (5) into Equation (6), we can obtain that

(7) $$k\sigma D_{min}^{3}-8.8V_{noise}\;D_{min}^{2}-32.4V_{noise}\;tD_{min}-17V_{noise}\;t^{2}=0$$

The above equation reveals that the data storage density limit of the TEDS is determined by three parameters, including σ, *t*, and *V*_noise_. Here, the value of σ is set to be 1 µC m^−2^, which was experimentally measured in previously reported works.^28^, ^29^, ^36^ Given three values of signal noise, i.e., *V*_noise_ =  0.1, 0.05, and 0.01 mV, the variation of *D*_min_ as a function of *t* is presented in Figure 5f by solving Equation (7). From the results in Figure 5f, we can conclude that reducing the values of *t* and *V*_noise_ can yield small *D*_min_. For the data acquisition system in this work (*V*_noise_= 0.1 mV for Keithley 6514), the *D*_min_ of 130 nm can be obtained when the thickness of electrification layer (*t*) is 10 nm. The corresponding theoretically estimated data storage density is derived to be 38.2 Gbit in.^−2^, which is even superior to recently emerged technology such as Blu‐ray Disc.^37^, ^38^ The storage density can be further enhanced given that the signal can be acquired at a smaller noise.

To visualize the data reading process, two kinds of computer software interface are programed according to the aforementioned algorithm in order to retrieve the data in real time. The first is a MATLAB program for reading the data in the disc‐shaped TEDS in **Figure** **6**a. From the software interface, we obtain a byte matrix. Each column of the byte matrix consists of 8 binary bits that represents a character. For example, the leftmost column is “01000100,” representing a letter “D.” For the same reasoning, the entire byte matrix represents a phrase “Data Storage,” as shown in the popped‐up window. Another software interface is realized by LABVIEW program. We can see from Figure 6b that the program identifies binary bits “0” and “1” from the voltage signal, as indicated by the state “off” and “on” of the Boole lights. Once the scanning completes, a character string “BINN” and its corresponding complete voltage signal are displayed on the software interface. The live process of the data reading can be found in Movie S1 (Supporting Information).

**Figure 6:**
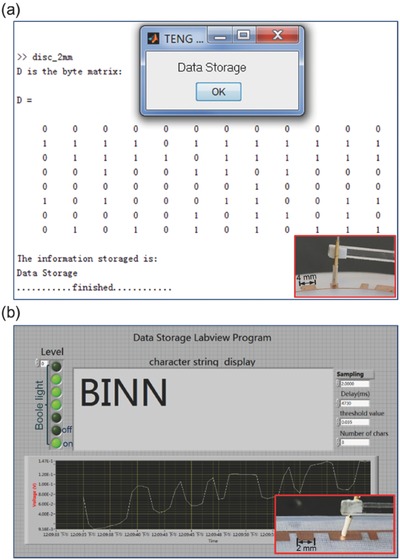
Demonstration of data reading process in real time. a) Data reading realized by MATLAB program on the disc‐shaped TEDS with the unit pattern size of 2 mm. b) Data reading realized by LABVIEW program on the strip‐shaped TEDS with the unit pattern size of 2 mm.

In summary, we have proposed a new data storage method based on triboelectrification‐induced voltage variation, which exhibits features, such as low power consumption, low cost, ultrathin flexible structure, easy fabrication, and high storage density. We successfully realized the writing of meaning sentences on the strip‐shaped and the disc‐shaped TEDS with the unit pattern size as small as 1 mm. Demonstrations of real‐time data retrieval are realized by MATLAB and LABVIEW. By theory simulation via COMSOL and theoretical analysis, three factors that determine the data storage density were investigated, including the surface charge density, the thickness of the electrification layer, and the background noise of acquired signal. Therefore, this work paves a new path to a fundamentally unique approach to high‐density data storage that may have widespread applications.

## Experimental Section

*Fabrication of the TEDS*: (1) Fabrication of the surface patterns. A layer of PMMA of 1 mm thickness was cut into 15 × 200 mm as a substrate. The substrate was coated with a layer of PTFE with dimensions of 15 × 200 × 0.06 mm. A mask made of polyethylene terephthalate was then prepared by laser cutting, on which information patterns were created in the form of hollow windows. After the mask was pasted on the substrate, a layer of copper was deposited onto the mask by magnetron sputtering. Upon removing the mask, the metal‐based surface patterns were left on the substrate. (2) Fabrication of the probe. A copper cylinder of 0.4 mm in diameter and 10 mm in height was adhered onto a mechanical arm. The copper cylinder was coated with an electrification layer (PTFE) of 0.06 mm in thickness. The mechanical arm was connected fixed on an electric motor that drives the probe scanning across the patterns either in linear or circular motion.

*COMSOL Simulation*: As depicted in Figure 2b, the dimensions of the PTFE layer on the substrate were set to be 10 × 2 × 0.05 mm. The metal line that connects individual patterns was set to be 9 × 0.1 × 0.005 mm. The length of the unit pattern and the unit interval was set to be 1 mm. The surface charge density on the electrification layer of the probe was set to be 4  × 10 ^− 8^ C m ^− 2^.

## Conflict of Interest

The authors declare no conflict of interest.

## Supporting information

Supplementary

Supplementary
