# Supplementary material for: Triboelectrification‐Enabled Self‐Powered Data Storage
Source: Adv Sci (Weinh). 2018 Jan 5;5(2):1700658. doi: 10.1002/advs.201700658 (PMC5826983; doi:10.1002/advs.201700658)
Supplement: Supplementary file 1 — Supplementary [file ADVS-5-1700658-s002.pdf]

Copyright WILEY-VCH Verlag GmbH & Co. KGaA, 69469 Weinheim, Germany, 2017.

ADVANCED SCIENCE Open Access

# Supporting Information

for *Adv. Sci.,* DOI: 10.1002/advs.201700658

# Triboelectrification-Enabled Self-Powered Data Storage

*Shuang Yang Kuang, Guang Zhu,* and Zhong Lin Wang**

Copyright WILEY-VCH Verlag GmbH & Co. KGaA, 69469 Weinheim, Germany, 2016.

# Supporting Information

## Triboelectrification-enabled self-powered data storage

*Shuang Yang Kuang, Guang Zhu*, Zhong Lin Wang**

Contents:

**Note S1:** The detailed derivation process of the electric potential between the metal patterns and the probe.

**Note S2:** The fitting process of the simulated curves in Figure 5b, c and d.

**Movie S1:** Demonstration of data reading on a disc-shaped TEDS by LABVIEW program.

## Note S1

## The detailed derivation process of the electric potential between the metal patterns and the probe.

Condition 1: Square-shaped reading probe

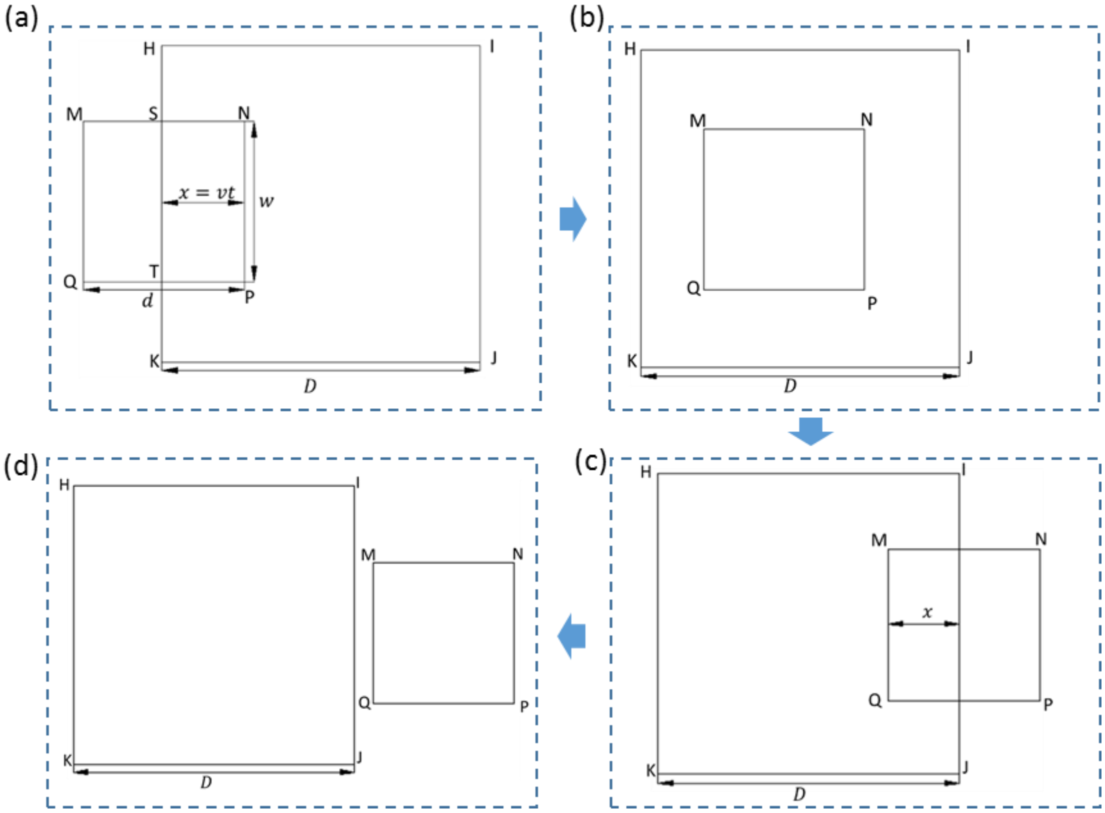


**Figure S1.** The process of data reading with square-shaped probe. (a) The state that the probe partially contacts with the metal pattern in the left side. (b) The state that the probe completely contacts with the metal pattern. (c) The state that the probe partially contacts with the metal pattern in the right side. (d) The state that the probe does not contact with the metal pattern.

Now, we deduce the analytical expression of the electric potential difference between metal probe and metal pattern. As illustrated in **Figure S1**a, Rectangle HIJK is unit metal pattern representing binary bit “0”. $D$ is the length of the unit pattern. Rectangle MNPQ is the cross section of the probe, representing the contact area between the electrification layer and the storage medium, $w$ and $d$ are its side length. SNPT is the contact area between the probe and the metal pattern. The thickness of the electrification layer $t_{thickness}$ is much smaller than $d$.

Now, we have $x = vt$, $v$ is sliding velocity of the probe, $t$ is the time, the initial instant t=0 represents the borderline NP overlapping with the borderline HK. After full contact between the probe and pattern, total charge Q has transferred from metal pattern to electrification layer, thus we have the initial surface charge density on the electrification layer $\sigma_0 = \frac{Q}{wd}$

(1), state in Figure S1a.

When$0 \leq x = vt \leq d, 0 \leq t \leq d/v$, the area of section MSTQ is $S_{MSTQ} = w(d - x)$

The charge on MSTQ is $Q' = \sigma_0 S_{MSTQ} = Q\left(1 - \frac{x}{d}\right)$

The charges on SNPT are screened by metal pattern, so the real charge density on MNPQ

is: $\sigma' = \frac{Q'}{wd} = \sigma_0\left(1 - \frac{v}{d}t\right)$

On the open-circuit condition and the condition of $t_{thickness}/d \to 0$, the electric potential difference $V_{oc}$ between the probe and the pattern is proportional to the real surface charge density,

$V_{OC} = -c\sigma' + V_0 = c\sigma_0\left(\frac{v}{d}t - 1\right) + V_0$, $0 \leq t \leq d/v$

where $c$ is a proportional constant. $V_0$ is the reference voltage.

At the time $= d/v$ ,

$V_{OC} = V_0$

(2), state in Figure S1b.

When $d \leq x = vt \leq D, d/v \leq t \leq D/v,\ S_{MSTQ} = 0,$ the charges on MNPQ are completely screened by metal pattern, thus we have

$V_{OC} = V_0, d/v \leq t \leq D/v$

(3), when $D/v \leq t \leq (D + d)/v$, unscreened surface appears at the right side, its area change from 0 to $wd$, which is symmetrical to the time interval $0 \leq t \leq d/v$. It's easy to get

$V_{OC} = c\sigma_0\left(\frac{D}{d} - \frac{v}{d}t\right) + V_0$

(4), When $x \geq D + d, t \geq (D + d)/v$, the unscreened surface area is the whole rectangular area MNPQ, it lasts for a time interval to the time when reading probe contacts with another metal pattern, thus we have

$\sigma' = \sigma_0, V_{OC} = -c\sigma_0 + V_0$

The $V_{oc}$ variation can be summarized in a formula:

$$V_{OC} = \begin{cases} c\sigma_0\left(\frac{v}{d}t - 1\right) + V_0 & ,0 < t \leq \frac{d}{v} \\ V_0 & ,\frac{d}{v} < t \leq \frac{D}{v} \\ c\sigma_0\left(\frac{D}{d} - \frac{v}{d}t\right) + V_0 & ,\frac{D}{v} < t \leq \frac{D+d}{v} \\ -c\sigma_0 + V_0 & ,t > \frac{D+d}{v} \end{cases} \qquad (S1)$$

Assuming that $c\sigma_0 = -0.1$ V, $V_0 = 0$ V, $d = 0.5$ mm, $v = 10$ mm/s, $D = 1$ mm, the corresponding analytical results are shown in Figure S2a,

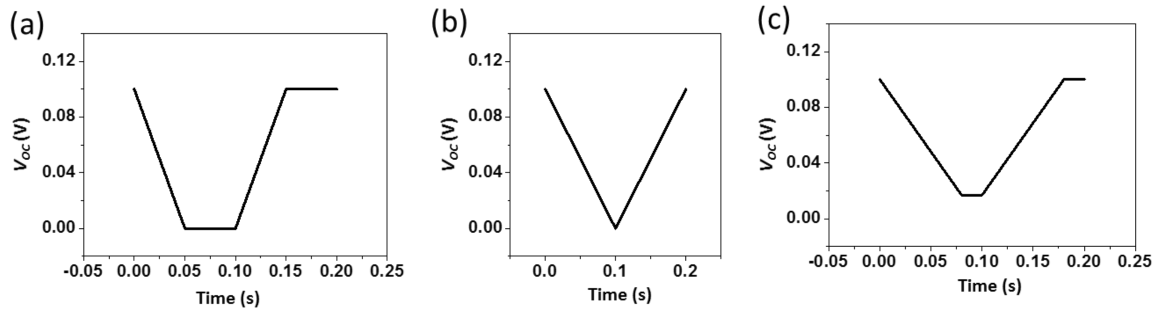

**Figure S2.** The plotted curve of equation (S1) at different parameter values. (A) At the parameter condition of $d = 0.5\ \mathrm{mm} < D = 1\ \mathrm{mm}$. (B) At the parameter condition of $d = D = 1\ \mathrm{mm}$. (C) At the parameter condition of $d = 1.2\ \mathrm{mm} > D = 1\ \mathrm{mm}$.

When $0 < d < D$, for example $d = 0.5\ \mathrm{mm} < D = 1\ \mathrm{mm}$, a sloped quasi-square wave can be observed, as shown in **Figure S2**a. As deduced in the main text, the amplitude of the wave is $H = -c\sigma_0$; the time span of the wave trough is $S = (D - d)/v$. When $d = D$ or $D < d < 2D$, $H$ and $S$ change, that yields the different curves in Figure S2b and c, respectively.

The three curves in Figure S2 show different characteristics, before discussing their applicability in data storage we need to discuss an important parameter named reading period, which is a time interval between two adjacent bits when reading. First we define some proper names. Take the curve in Figure S2a as example, the rising edge is the curve from the end of the trough to the start of the adjacent crest, the falling edge is the curve from the end of the crest to the start of the adjacent trough. We define that the reading period $T$ is the total time from the start of the rising edge to the end of the adjacent crest. Based on this definitions we can easy know from the $V_{\mathrm{oc}}$ expression that $T = \frac{d}{v} + \left(\frac{D}{v} - \frac{d}{v}\right) = D/v$. We define another parameter-time ratio $Tr$, which is the ratio between the rising edge and the adjacent crest in time axis: $Tr = \frac{d}{v} : \left(\frac{D}{v} - \frac{d}{v}\right) = \frac{d}{D-d}$. When $Tr$=0, the voltage signal is a standard square wave. When $d = D/2, Tr = 1$, it means the rising edge is equal to the crest on time axis. When $Tr > 1$, that means the rising edge is wider on time axis than the crest, this situation will decrease the accuracy rate of the reading program, because our reading program identifies binary bit "0" and "1" by acquiring the voltage value in the crest and trough periodically, the much shorter width of crest and trough will improve the probability to acquire the value at the rising edge or the falling edge. So the $V_{\mathrm{oc}}$ curves in Figure S2b ($Tr = +\infty$) and Figure S2c ($Tr > 1$) are not ideal curves for data storage. Technically, we restrict the size of the reading probe to meet relation $0 < Tr \leq 1, d \leq D/2$, *i.e.* the situation shown in Figure S2a.

Condition 2 : Round-shaped reading probe

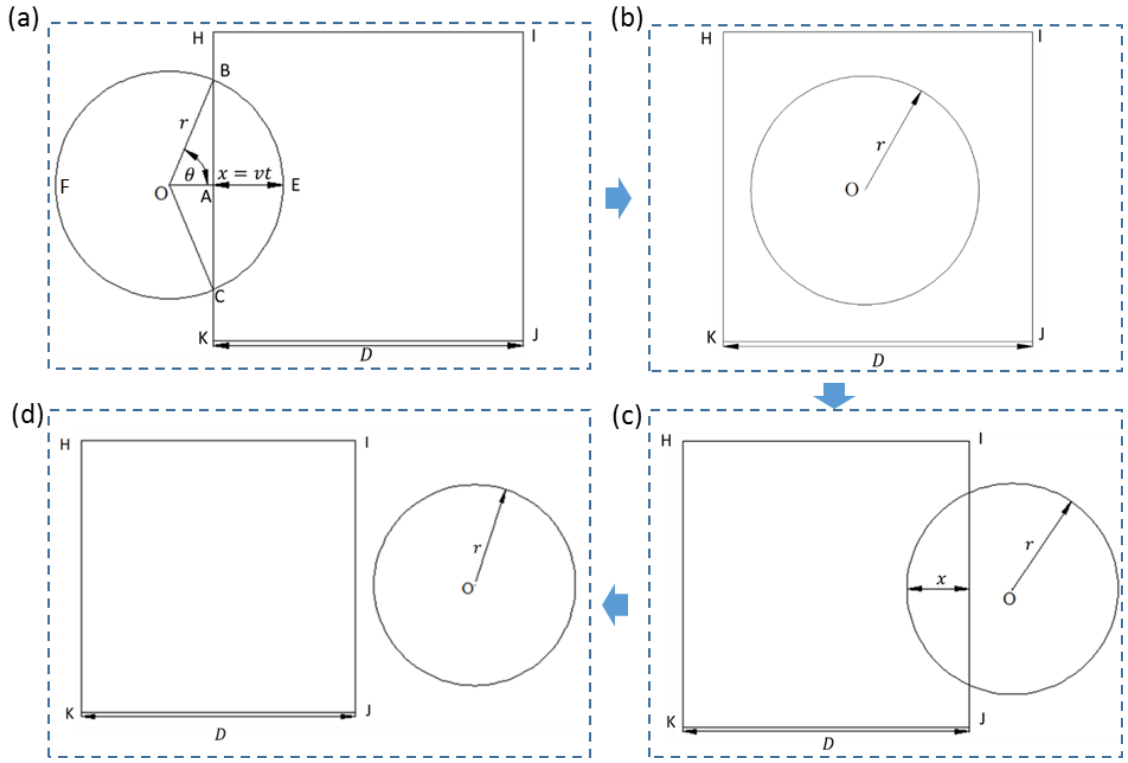


**Figure S3.** The process of data reading with round-shaped probe. (a) The state that the probe partially contacts with the metal pattern in the left side. (b) The state that the probe completely contacts with the metal pattern. (c) The state that the probe partially contacts with the metal pattern in the right side. (d) The state that the probe does not contact with the metal pattern.

For this situation, we have the same deduced steps like in condition 1. Rectangle HIJK is unit metal pattern. $D$ is the length of the pattern. Circle O is the cross section of the probe, $r$ is its radius. BEC is the contact area between the probe and the pattern.

Like in condition 1, we have $OB = r, AE = x = vt, \sigma_0 = \frac{Q}{\pi r^2}$

(1), state in **Figure S3**a.

When $0 \le x \le r, 0 \le t \le r/v, OA = r - x$

$$AB = \sqrt{OB^2 - OA^2} = \sqrt{2rx - x^2}, cos\theta = \frac{OA}{OB} = 1 - \frac{x}{r}, \theta = \arccos\left(1 - \frac{x}{r}\right)$$

The area of triangle OBC and sector OBEC is:

$$S_{\triangle OBC} = AB * OA = (r-x)\sqrt{2rx-x^2}, S_{OBEC} = \frac{1}{2}r * r * 2\theta = r^2 \arccos\left(1-\frac{x}{r}\right)$$

So the area of the covered surface BEC is:

$$S_{BEC} = S_{OBEC} - S_{\triangle OBC} = r^2 \arccos\left(1-\frac{x}{r}\right) - (r-x)\sqrt{2rx-x^2}$$

Lastly the uncovered surface area is:

$$S = \pi r^2 - S_{BEC} = \pi r^2 + (r-x)\sqrt{2rx-x^2} - r^2 \arccos\left(1-\frac{x}{r}\right)$$

Uncovered surface area charge: $Q' = S\sigma_0 = QS/\pi r^2$

Real surface charge density of electrification layer:

$$\sigma' = \frac{Q\prime}{\pi r^2} = \sigma_0[1 + \frac{r-x}{\pi r^2}\sqrt{2rx-x^2} - \frac{1}{\pi}\arccos\left(1-\frac{x}{r}\right)]$$

electric potential difference $V_{oc}$ :

$$V_{OC} = -c\sigma' + V_0 = k\sigma_0[\frac{1}{\pi}\arccos\left(1-\frac{x}{r}\right) - \frac{r-x}{\pi r^2}\sqrt{2rx-x^2} - 1] + V_0$$

When $r < x < 2r, r/v < t < 2r/v, OA = x - r,$

$$cos\theta = \frac{OA}{OB} = \frac{x}{r} - 1, \theta = \arccos\left(\frac{x}{r} - 1\right)$$

$$S_{\triangle OBC} = AB * OA = (x - r\ )\sqrt{2rx-x^2}, S_{OBFC} = \frac{1}{2}r * r * 2\theta = r^2 \arccos\left(\frac{x}{r} - 1\right)$$

$$S = S_{OBFC} - S_{\triangle OBC} = r^2 \arccos\left(\frac{x}{r} - 1\right) - (x-r)\sqrt{2rx-x^2}$$

$$\sigma' = \frac{S\sigma_0}{\pi r^2} = \sigma_0[\frac{1}{\pi}\arccos\left(\frac{x}{r} - 1\right) - \frac{x-r}{\pi r^2}\sqrt{2rx-x^2}]$$

$$V_{OC} = -k\sigma' + V_0 = c\sigma_0[\frac{x-r}{\pi r^2}\sqrt{2rx-x^2} - \frac{1}{\pi}\arccos\left(\frac{x}{r} - 1\right)] + V_0$$

At the position of $x = 2r, V_{OC} = V_0, t = d/v$

(2), state in Figure S3b.

When $2r \le x \le D, 2r/v < t < D/v$, the surface charges are totally screened by metal pattern, $\sigma' = 0, V_{OC} = V_0$

(3), When $D \le x \le D + 2r, D/v < t < (D + 2r)/v$, unscreened surface charges appear at the right side, its area changes from 0 to $\pi r^2$, which is symmetrical to the time interval $0 \le t \le 2r/v$, so we get this two symmetrical conditions,

When $D \le x \le D + r, D/r < t < (D + r)/v$,

$$V_{OC} = c\sigma_0[\frac{r - x + D}{\pi r^2}\sqrt{2r(x - D) - (x - D)^2} - \frac{1}{\pi}\arccos\left(1 - \frac{x - D}{r}\right)] + V_0$$

When $D + r \le x \le D + 2r, (D + r)/v < t < (D + 2r)/v$,

$$V_{OC} = c\sigma_0[\frac{1}{\pi}\arccos\left(\frac{x-D}{r} - 1\right) - \frac{x-D-r}{\pi r^2}\sqrt{2r(x - D) - (x - D)^2} - 1] + V_0$$

(4), state in Figure S3d.

When $x \ge D + 2r, t \ge (D + 2r)/v$, unscreened surface area is the whole circular area $\pi r^2$, it lasts for a time interval to the time when reading probe contacts another metal pattern. We easily get $V_{oc}$ expression at this time interval: $\sigma' = \sigma_0, V_{OC} = -c\sigma_0 + V_0$ .

Lastly, the $V_{oc}$ variation can be summarized in a formula:

$$V_{OC} = \begin{cases} c\sigma_0[\frac{1}{\pi}\arccos\left(1 - \frac{x}{r}\right) - \frac{r - x}{\pi r^2}\sqrt{2rx - x^2} - 1] + V_0 & ,0 < t \le r/v \\ c\sigma_0\left[\frac{x - r}{\pi r^2}\sqrt{2rx - x^2} - \frac{1}{\pi}\arccos\left(\frac{x}{r} - 1\right)\right] + V_0 & ,r/v < t \le 2r/v \\ V_0 & ,2r/v < t \le D/v \\ c\sigma_0\left[\frac{r + D - x}{\pi r^2}\sqrt{2r(x - D) - (x - D)^2} - \frac{1}{\pi}\arccos\left(1 - \frac{x - D}{r}\right)\right] + V_0 & ,\frac{D}{v} < t \le \frac{D + r}{v} \\ c\sigma_0[\frac{1}{\pi}\arccos\left(\frac{x - D}{r} - 1\right) - \frac{x - D - r}{\pi r^2}\sqrt{2r(x - D) - (x - D)^2} - 1] + V_0 & ,\frac{D + r}{v} < t \le \frac{D + 2r}{v} \\ -c\sigma_0 + V_0 & ,t > \frac{D + 2r}{v} \end{cases}$$

(S2)

Assuming that $c\sigma_0 = -0.1$ V, $V_0 = 0$ V, $r = 0.25$ mm, $v = 10$ mm/s, $D = 1$ mm, the corresponding analytical result is shown in Figure S4.

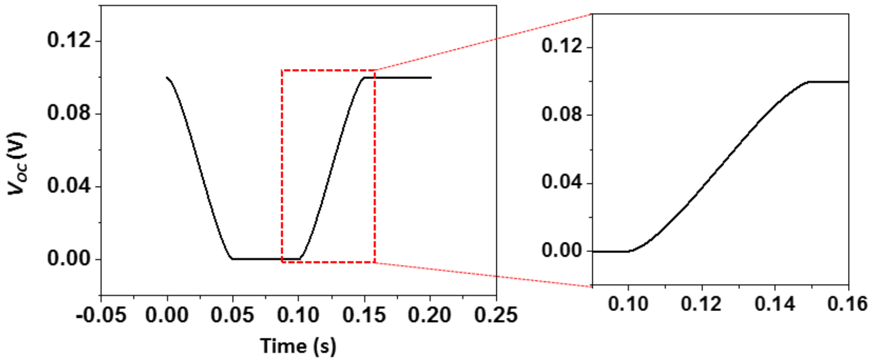


**Figure S4.** The plotted curve of equation (S2) at the given parameter value above.

It can be obviously observed that there is one big difference at the rising edge and falling edge between the two conditions. In condition 1, the rising edge and falling edge are both straight lines, and the edge of the crest or trough is sharpened (not differentiable). In condition 2, the rising edge and falling edge are not straight lines and the edge of the crest or trough becomes rounded (differentiable). Take the rising edge as an example, the absolute slope of the curve increases from zero (at the end of trough) to maximum (at the center of the falling edge), then decreases from maximum to zero (at the start of the crest). But the slope of rising edge or fallowing edge for the curve in condition 1 keeps unchanged. This difference comes from the different shape of the reading probe.

**Note S2**

**The fitting process of the simulated curves in Figure 5b, c and d.**

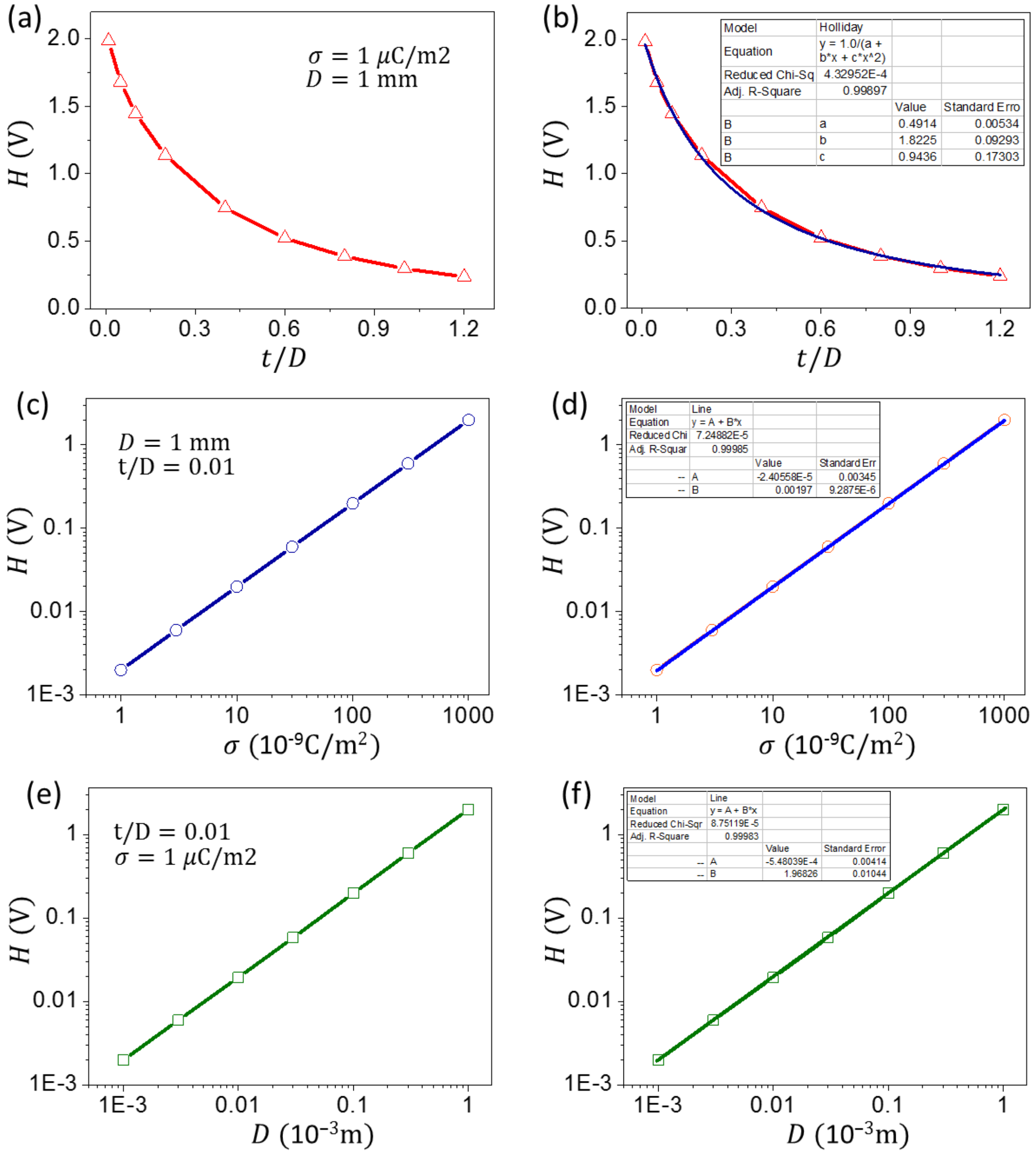


**Figure S5.** The fitting process of the curves in Figure 5 in the main text. (a) The simulated result of the relation between $H$ and $t$ at the certain values of $D$ and $\sigma$. (b) The fitting result of data in Figure S5a via Origin software. (c) The simulated result of the relation between $H$ and $\sigma$ at the certain values of $D$ and $t/D$. (d) The fitting result of data in Figure S5c via Origin software. (e) The simulated result of the relation between $H$ and $D$ at the certain values of $\sigma$ and $t/D$. (f) The fitting result of data in Figure S5e via Origin software.

Figure S5a, c and e are the simulated curves at different parameter values, which has been detailed clarified in the main text. Figure S5b, d and f are the fitting results of the corresponding curves. The fitted parameters can be seen in the inset of the figures.
